# Optimization of a group‐based intervention for people living with severe obesity (PROGROUP): Understanding fidelity to delivery and the patient experience

**DOI:** 10.1111/bjhp.70051

**Published:** 2026-02-02

**Authors:** Lily Hawkins, Mark Tarrant, Shokraneh Moghadam, Dawn Swancutt, Rod Sheaff, Laura Hollands, Jonathan Pinkney, Jenny Lloyd

**Affiliations:** ^1^ Faculty of Health and Life Sciences University of Exeter Exeter UK; ^2^ Faculty of Health University of Plymouth Plymouth UK; ^3^ Present address: NHS Greater Glasgow and Clyde Glasgow UK

**Keywords:** fidelity, obesity, patient experience, social identity

## Abstract

**Objectives:**

Understanding the fidelity of delivery of complex health behaviour interventions is crucial in determining their effectiveness and identifying aspects needing refinement. PROGROUP is a group‐based intervention for people with severe obesity. It aims to promote a shared social identity to support behaviour change. Data from a feasibility randomized controlled trial (fRCT) were used to assess fidelity of intervention delivery and the impact on patient experiences, to optimize the intervention for a main trial.

**Methods:**

Data from 18 patient and five facilitator interviews, audio and video data of group sessions, two fidelity checklists, support calls and a group processes questionnaire were used to assess fidelity of delivery to intervention principles, patients' experience of the intervention and areas for optimization.

**Results:**

The number of activities delivered and facilitator confidence and rapport with the group affected fidelity to intervention principles. The facilitators' delivery style, group composition and attendance affected the groups' sense of social identity. Accordingly, the intervention content was revised to ensure better balance between educational material and group activities, to increase facilitator confidence and enable flexible delivery.

**Conclusions:**

The success of group‐based interventions relies on the facilitator addressing the group's needs and creating conditions for a shared social identity to develop. Assessment of fidelity to the manual content and core function of PROGROUP enabled identification of components needing refinement, incorporating both facilitator and patient perspectives. The assessment and optimization process offer a blueprint for evaluating other group‐based interventions.

**Statement of Contribution**

What is already known?

- Understanding fidelity of delivery of complex interventions is crucial in testing the causal pathways of an intervention and highlighting areas for improvement.
- Fidelity assessments tend to focus on delivery. Other elements are poorly assessed.
- There is little evidence documenting how to use fidelity data to inform optimization of group‐based interventions.

What does this study add?

- Knowledge of how to optimize a group‐based behavioural intervention based on patients' experience, as well as fidelity to delivery data.
- A method to optimize the function of group‐based behavioural interventions through the assessment of fidelity to form.

## INTRODUCTION

Complex interventions are widely used in health services for behavioural management of health issues such as obesity and diabetes. Conventionally defined as interventions with several interacting components, such interventions present several challenges for evaluators (Lewin et al., 2017). These challenges include difficulty in standardizing intervention design and delivery, their sensitivity to features of the setting in which they are delivered, and the length and complexity of causal chains linking intervention with outcomes. These causal chains represent the mechanisms through which an intervention is assumed to work, developed through initially reviewing the literature and modelling processes (Craig et al., 2008; Moore et al., 2015; Skivington et al., 2021), before testing these causal pathways in the piloting and feasibility phases to see if they are working as intended. Finally, useful or redundant components are identified and refinements made to the intervention in advance of the full evaluation (Levati et al., 2016; McCrabb et al., 2020; Skivington et al.). Part of this testing should involve assessing whether the intervention has been delivered as designed (i.e. with fidelity). Not only should this involve assessing whether intervention components were delivered (fidelity to form), but also whether the intervention achieves its core function, (fidelity to function) (Esmail et al., 2020; Hill et al., 2020; Mars et al., 2013; Toomey et al., 2020; Walton et al., 2017).

Data collection methods to assess delivery fidelity include self‐reported checklists, observations, questionnaires and interviewing those delivering and receiving the intervention. The recipient perspective provides essential data on how the intervention is experienced and whether this aligns with its core purpose (O'Cathain et al., 2019). When misaligned, evaluators can assess whether this is due to poor fidelity to form and/or a flaw in the design of the intervention. These data can then inform changes necessary to optimize the intervention.

This paper aims to present the optimization process of the PROGROUP intervention using fidelity to delivery data collected as part of a feasibility Randomized Control Trial (fRCT: ISRCTN number 22088800). The fRCT (Swancutt et al., 2022) took place between June 2022 and August 2023 across three UK NHS sites (Midlands, Wales and South West), involving 94 patients (47 PROGROUP, in four groups, and 47 Usual Care). Facilitator training optimization is reported elsewhere (Moghadam et al., in prep).

### The PROGROUP intervention

PROGROUP is a 15‐session (12 group‐based, 3 individual) intervention designed to support lifestyle changes for people living with severe obesity for delivery by health care professionals in specialist weight management services. Its assumed mechanisms of action are based on the Social Identity Model of Behaviour Change (Tarrant et al., 2020). This model argues that the group processes arising during delivery (e.g. shared values and social support) give opportunities to create a shared social identity, allowing the intervention group to become a resource underpinning its members' motivation and capability (e.g. self‐efficacy and knowledge) to engage in behaviour change. The core function of the PROGROUP intervention is to create this sense of shared social identity amongst patients in the group. To do this, intervention components have been designed with this function in mind. This is referred to as the ‘form’ of PROGROUP, which involves delivering core behaviour change content via structured group activities according to a set of delivery principles (the PROGROUP principles—see Supporting Information S1; see Moghadam et al. (2024) for a report of the intervention development). Whilst PROGROUP activities have been manualized, it is crucial they are delivered flexibly and according to the PROGROUP principles to achieve their function. In group‐based interventions, the social processes involved as members and facilitators connect and start to form a shared social identity increase the complexity of how causal chains affect desired outcomes. Assessing these processes is challenging, but essential in order to understand and develop a group programme that can support behaviour change.

## METHOD

### Participants

Facilitators (*n* = 5) and group members (*n* = 47) provided mixed methods data at different time points across the fRCT. Facilitators were health care practitioners in specialist weight management services. Patients in the intervention had a mean age of 49.26 years old (range: 26.2–72.3 years old); 68% (*n* = 32) were female; and 84% (*n* = 36) were white British.

### Data collection

**FIGURE 1:**
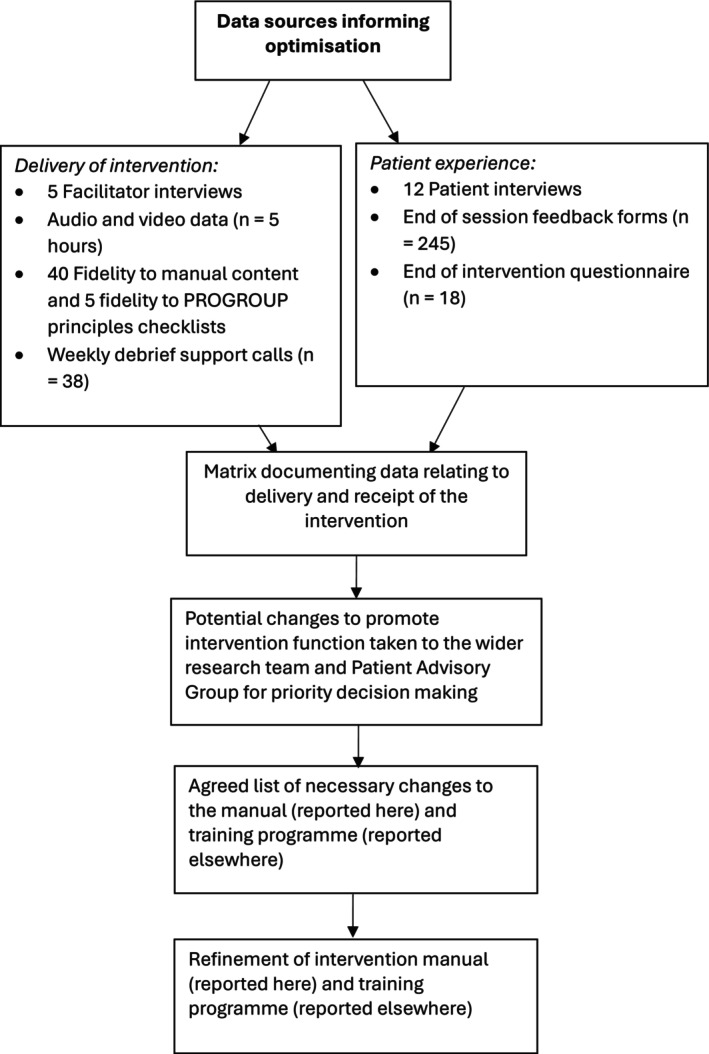
Optimization process of the PROGROUP intervention.

Data sources used to assess fidelity of delivery and patient experience are represented in Figure 1. Informed consent was obtained from all trial and interview participants (see Swancutt et al., 2022 for details of trial consent procedure).

### Intervention delivery

To assess fidelity to the manual content, facilitators completed a checklist (see Supporting Information S2) after each group session, specifying whether activities had been delivered fully or partially, including reasons for any partial delivery. The accuracy of this self‐reported checklist was assessed against a researcher‐completed checklist for seven in‐person observations across sites. To assess fidelity to the PROGROUP principles, five researchers (LH, MT, SM, RW, JL) used selected video/audio data (sessions 1, 2, 9 and 11) from two sites (Southwest and Wales) and completed a checklist (Supporting Information S3). These observations were discussed within the team and notes synthesized, highlighting necessary refinements to the intervention manual (reported here) and training programme (reported separately).

To understand their experience of delivery, one semi‐structured interview was conducted with each facilitator post‐intervention delivery (*n* = 5) (Supporting Information S4a); weekly support calls with a researcher were offered to address any queries or concerns and used to corroborate data provided within interviews (see Analysis section).

### Intervention receipt

Immediately after each group session, patients completed a short feedback form to assess satisfaction and perceived group connection (Supporting Information S5a). Following group sessions 2, 7 and 12, patients were sent the Group Processes Questionnaire (Supporting Information S6), including measures to capture changes in social identification with other members of the group and social support (Haslam et al., 2005; Postmes et al., 2013). Semi‐structured interviews post‐intervention (*n* = 12) were conducted for a purposive sample of patients, (based on age, gender and attendance) across sites (Supporting Information S4b).

Free text comments and suggestions from the patient ‘end of intervention’ questionnaire (Supporting Information S5b) relating to how PROGROUP could be improved were collated and informed the optimization process.

### Analysis

#### Quantitative analysis

Means and standard deviations, or frequencies and percentages where appropriate, were calculated for each of the measures comprising the patient end of session feedback forms and group processes questionnaires. Data from the end of session feedback for each group were averaged to depict how members' connection to other group members developed over time.

For the fidelity to form checklist, frequency and percentages were calculated for the activities reported as fully delivered, in each session, across each site. A mean percentage across all group sessions, as well as across sites and each session was then calculated, to give an overall summary of how much content was delivered across sites, for each session and in total across the programme.

#### Qualitative analysis

Qualitative data were uploaded to NVivo (QSR International; Version 14 Pro). Taking both an inductive and deductive thematic approach to analysis (Braun & Clarke, 2023), two coding frameworks were developed to categorize the data from (i) PROGROUP patient interview transcripts and any free text data from the end of programme questionnaire and (ii) facilitator interview transcripts, free text data on the fidelity to manual content checklist and support call notes. Interviews were transcribed using targeted transcription, to facilitate rapid identification of data related to feasibility and acceptability of the intervention. For the interview data, five researchers (LH, RW, SM, DS, JL) independently read and coded line‐by‐line four patient and two facilitator interview transcripts and discussed their coding to develop and agree the coding framework for each group, which included combining codes and arranging them into higher order categories. The agreed frameworks were then used by LH, RW and SM to code the remaining interviews, with new inductive codes added as appropriate. While detailed codes were developed inductively, the categories were more deductive, reflecting each interview topic guide (Supporting Information S4). Attention was paid to negative or deviant cases to inform developing themes and interpretation. Notes from support calls and free text data from questionnaires were incorporated into the facilitator and patient frameworks.

### Optimization process

Qualitative and quantitative data were integrated and triangulated using a convergence coding matrix to display key themes and findings related to delivery and receipt of the intervention across data sources (Supporting Information S7). Patient experiences of receiving the intervention, reasons for compromised delivery fidelity and potential adaptations to achieve intervention function were discussed with the research team and our Patient Advisory Group over several meetings, to decide on changes to be made (see Optimization in Results for changes).

## RESULTS

### Delivery of the intervention

Overall, much of the intervention was delivered flexibly and according to the PROGROUP principles. However, facilitators' use of flexible delivery and the PROGROUP principles varied, resulting in differences in fidelity to function across groups. Reasons for this variation included three interlinked factors: the number of activities facilitators had to cover each session, facilitator confidence and their rapport with the group. Quotes from interviews are provided in text (using identifiers F = facilitator and P = patient with assigned study number, e.g. P1000), with further supporting quotes relating to delivery by facilitators and patient experience in Supporting Information S8 (Data S1.1–S1.6 and S2.1–S2.11).

Researcher observations, video and audio data showed that facilitators delivered the intervention according to the PROGROUP principles and delivered the majority of the manualized activities (68% delivered across all groups). However, there were some inconsistencies across sessions and between facilitators. For example, data from interviews and support calls revealed that all facilitators felt that there were too many activities to deliver in the allotted time, especially in early sessions (e.g. Supporting Information S8: Data S1.1 and S1.2). For some facilitators, there was also uncertainty around when and how to deliver PROGROUP activities flexibly to address group needs. These facilitators reported trying to cover all the manual activities, leading to delivery that was sometimes rushed and didactic.

what happened was I just ended up going into like delivery, or teacher mode almost, which I know personally as a facilitator like it's not that helpful to have someone tell you stuff for an hour and a half. F01 [Facilitator01]

Video data revealed that in these instances, facilitators failed to deliver according to the PROGROUP principles, especially that of highlighting similarities between group members, which was a key activity for building connection. Furthermore, fidelity to manual content checklists revealed that these time pressures often led to facilitators missing or only partially delivering activities that promoted a positive group atmosphere, for example, the ‘group pulse’, designed to gain feedback on how sessions were going, connect group members, encourage support and promote a shared social identity. These pressures therefore created missed opportunities to build a sense of shared social identity, which is essential for achieving desired outcomes.

There was, however, evidence from session observations and facilitator interviews that some facilitators did deliver according to the PROGROUP principles, applying the content flexibly to address the needs of the group.

although I delivered the majority of the content, it wasn't a rigid thing in my head, it was a there are the things that we need to cover, but actually the space in the sessions to be able to kind of go a little bit off piste if somebody kinda took it that way and I felt like that was well received by them. F05

Facilitators' ability to deliver flexibly was linked to previous experience and greater confidence in group‐based delivery and understanding of when to take a flexible approach (Data S1.3)

I think we were all flexing the content a bit based on particular groups, so it's interesting…are there bits of the content that weren't delivered because [for] most groups it wasn't relevant or important, or is about saying there's a bulk of content here, based on what this group needs you can flex it a little bit. But yeah 100% I would say that that was to do with my experience and feeling confident to do that. F04

Researcher observations corroborated that a flexible delivery style was also prioritized by some facilitators in sessions towards the end of the programme, with some facilitators tailoring content towards the needs of the group and providing opportunities for patients to share, rather than delivering activities exactly as manualized. As expected, overall delivery of manual content for these facilitators was lower as they tailored these to the needs of the group. For example, fidelity to manual content checklists demonstrated that facilitators prioritized activities designed to promote a sense of shared social identity (e.g. social network mapping) and flexed or omitted content which was less pertinent to the function of PROGROUP (e.g. physical activity).

The extent to which facilitators employed the PROGROUP principles and the confidence they felt in delivering flexibly was also linked to their relationship with their group. For example, video data and support calls indicated that facilitators who were more confident in delivering flexibly, tended to also have a better rapport with their group and be more proactive in using the PROGROUP principles and flexible delivery to focus on building a relationship with their group (Data S1.4). These facilitators also recognized their role in promoting a sense of shared social identity and used their knowledge of the group to do so. For example, in interviews, one facilitator reported actively engaging methods to prevent negative interactions, encourage attendance and sharing of experiences at sessions, to try to promote a sense of shared social identity (Data S1.5).

what I'm listening out for is anybody who wants to disclose something—it makes them a little bit a vulnerable, it comes a bit confessional—those topics, I'm very on top of it I'm kind of—I know my individuals in the room and I'm trying to catch it. F03

Video and audio data suggested that delivery based on employing PROGROUP principles strengthened the rapport between the facilitator and their group through their use of humour and conversational delivery style, resulting in greater patient engagement (demonstrated by greater discussion amongst patients and comments on the positivity of the group), creating a positive feedback loop that strengthened shared social identity.

By contrast, facilitators delivering with a more didactic and content focussed delivery style found it difficult to build a rapport, creating a negative feedback loop which reduced social connectedness. For example, in an interview, one facilitator reported that their group was quiet and less engaged, leading the facilitator to feel like the group was not supportive of them.

with [group 2]…I felt like there was a balance there and there was trust, whereas with [group 3], it definitely wasn't like that, I felt like I needed to deliver the content and actually they were thinking like you're not telling me anything I don't know, so it wasn't that supportive environment. F05

Notably, with the same group [3], a different facilitator's use of a ‘group pulse’ activity resulted in greater engagement from the group, which in turn promoted a positive group atmosphere and started the group connecting (Data S1.6). In an interview, this facilitator reported that they changed their delivery style over the course of the intervention programme, developing the confidence to deliver more according to the PROGROUP principles than didactically. In an interview, they reported that this helped to promote a sense of shared social identity and improved engagement and rapport with the group.

gradually as I've gone through, the timings have become less important with more of a focus on actually what the group are saying, how the group are interacting with each other, and bringing them through. F02

### Recipient perspective

Twelve patients (P) (9 female and 3 male; age range 30–66 years) were interviewed across the three sites (Southwest 3; Wales 2; Midlands 7). Response rate for the end of intervention questionnaire was 51% (*n* = 18/35).

Patients were generally positive about the intervention, with end of session feedback indicating they were satisfied with the programme (median score 4.5 out of 7 across sites, *n* = 245, 100% response rate for patients attending group sessions). However, as with facilitator delivery, patients' sense of shared social identity varied across groups and was affected by whether delivery of the intervention was in line with the individual and group needs, group composition and attendance.

Delivery style and use of the PROGROUP principles were key factors in whether the group felt a sense of connectedness. In the end of intervention feedback and interviews, some patients reported that the facilitators' delivery style helped to create a sense of safety, making sessions feel relaxed and comfortable, where it was easy to contribute (Data 2.1–2.3).

…he had time for everyone without any exception. You know? He treated everyone exactly the same way. So you felt assured and confident in approaching him and asking questions as you knew that he was genuinely listening and took an interest a genuine interest, you know? P3009

As reported for facilitator delivery, in interviews, moments of flexibility in delivery and allowing patients to go ‘off point’ to discuss issues between themselves were also highly valued by patients.

Listen really, and be supportive and recognise the little things that bothered each of us. In the group, I think we tended to get off point but those things I think were really important for individuals in the group and in that sort of sense it was like a mini counselling session, which I think really worked for the group, it really worked for me. P2052

Patients also reported key moments where a facilitator had a pivotal role in bringing the group together and promoting a sense of shared social identity, group connection and support.

I think it was [facilitator]—he said “you haven't got a group yet,” I'm like no and he was like “we're doing it today, you're thinking of a name,” so I think it was about 5 weeks in, very late, and we got it going and then we've been messaging, breaking the ice and making each other feel like we're there to support each other. I post recipes on there and other people post recipes and just generally have a lot of chit chat. P2052

However, while for some groups facilitator delivery promoted a sense of shared social identity, for other groups, delivery did not always align with the group's needs. There were some examples where patients felt that the content covered was not in line with their specific needs.

I wouldn't say it was information that most people wouldn't already know. Obviously, talking about your habits and things like that is always good to talk about, because you might recognise something that you're not realising you do, but I feel like the information they gave was a little bit school level, if that makes sense? Most people should already know these things. P1005

For other patients, input from dominant group members meant that there was no time to cover the content they wanted, indicating that facilitators did not always manage the group sufficiently to address individual needs equitably.

The people in the group just discussed the problems and issues they had over the years. The facilitators often did not get to cover the programme, so it was rushed and lacked the content I wanted. Participant, End of Intervention Feedback

**FIGURE 2:**
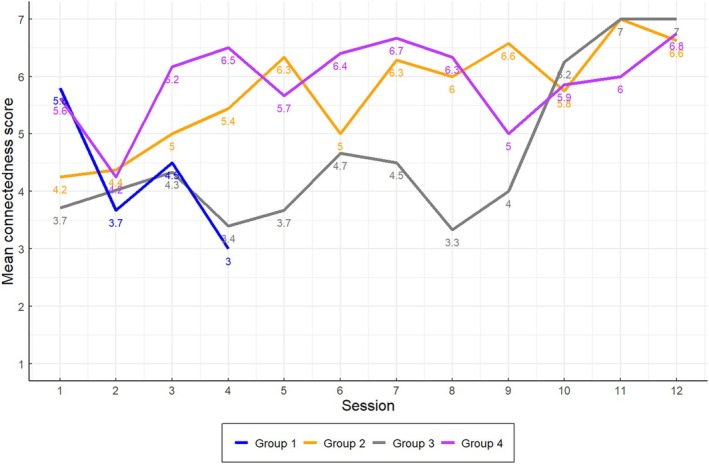
Mean patient group connectedness scores across the intervention period. Group 1 concluded after session 5 due to low attendance and data was not collected post‐session 4 due to lack of a group.

End of session data on patients' connection to other group members (rated on a scale of 0–7) also reflected these differences in group management. For some groups, patients felt strongly connected to the group after the first few weeks, whereas for other groups, where the group was not as actively managed, patients reported lower levels of connection, for a longer period of time (See Figure 2).

Patients reported that in groups where a sense of a shared social identity was weaker or took longer to develop, there were core opportunities where delivery could have improved the groups' connection to each other. One example cited by patients in end of intervention feedback was if contact with each other outside of sessions, or the use of a WhatsApp group, was emphasized much earlier within the programme, patients may have felt a greater sense of shared social identity with other group members (Data S2.4–S2.6).

Other factors as well as delivery also contributed to social identity development. Interview data revealed that for some patients, the initial composition of the group, including having people who they felt similar to and who understood what it was like to live with obesity, was important in being able to identify with the other group members. Importantly, where similarities existed within the group, this enabled a positive group atmosphere where patients felt understood, valued, like a ‘*team*’ and this helped patients foster a sense of support within the group (Data 2.7–2.9). These were also often the groups where flexible delivery by the facilitator had enabled discussions between group members.

It's so lovely because it's so nice to talk to people, outside of your family, who've got so much in common with yourselves. And I think in that scenario of it being in the group and you're all there with people that actually, you're saying things and they're like ‘yeah, we know where you're coming from’ … These people know how I feel, and I think it's been so beneficial, I think it's been great. P2031

In interviews, patients from these groups also reported changes in behaviour and thinking around relapse prevention and negative self‐talk.

I just thought, right okay well do you know what, I certainly enjoyed yesterday…you might have put a couple of pounds on ‐ well what the hell, and I just kind of showered, got my walking boots on and went out for a four‐mile walk….As you said I didn't beat myself up about it at all, you know, just said well just make sure you don't do that every day. P3009

However, for other groups, the composition of the group and sense of shared social identity was affected by diverse personalities and age groups, making it difficult for some group members to connect in the beginning.

Yes, different personalities and different age groups…we knew we were all there for the same reason. It was a bit awkward trying to gel; I was probably the oldest person there. P2054

This was corroborated by data from the Group Processes Questionnaire. For example, one group reported lower social identification (Group 3 (*n* = 21): M [Mean] = 4.61) and social support (Group 3: M = 4.67) compared with two other groups (Group 2 (*n* = 26): M social identification = 5.57, M social support = 5.32 and Group 4 (n = 21): M social identification = 5.75, M social support = 5.05). Response rates were low, however (61%, *n* = 111/126 across all groups and time points), so it was not possible to determine whether differences were significant, and these data should be interpreted with caution. See Discussion for further consideration of the low response rates.

Interview and end of intervention feedback indicated that this difference in sense of shared social identity was also affected by poor attendance and continuity of the group, and so, patients found it hard to ‘gel’ with each other (Data 2.10). Where attendance was inconsistent, in interviews, patients in these small and unstable groups reported that having different people in each session also meant that the facilitator spent time recapping content rather than spending time interacting and developing a sense of shared social identity.

Yeah you just can't gel with people if you're not seeing them enough do you know what I mean, and then you're having to re‐cap at the start because people have missed but then not everybody has it's just a shame do you know what I mean it's just we didn't feel close. P2057

These patients also suggested that if commitment and attendance at group sessions had been emphasized at the beginning of sessions, engagement with the intervention may have been improved (Data S2.11). End of intervention feedback from some patients also went further to highlight that low attendance negatively impacted motivation to engage in change.

I feel my group wasn't big enough, it folded quickly. Some of the people didn't seem to really understand why they were there. I have waited a lifetime for something like this group and I feel abandoned, depressed and [no] longer see me losing the weight and keeping it off. Participant, End of Intervention Feedback.

### Optimization

Patterns and inconsistencies in delivery and their impact on intervention function highlighted areas for improvement. These areas focussed on addressing the issues which hindered achieving the intervention function, with changes made to the intervention manual and associated resources (summarized in Table 1). To help facilitators to deliver in line with the function of the intervention and to aid facilitator confidence in delivering according to core intervention principles, changes included reducing the number of activities within sessions and developing and providing resources for use within manuals and training. To aid patient engagement, assigning group champions was introduced to encourage a sense of ownership over the group and connection on the areas that mattered to them the most. For details of the optimization process, see ‘Optimization process’ in Methods.

**TABLE 1:** Details of changes to the intervention in response to delivery fidelity findings.

Area to be optimized (data source)	Purpose of change	Modifications in response to findings
**Number of activities within sessions** (facilitator interviews; fidelity to manual content and fidelity to PROGROUP principles checklists; video and audio data)	Aimed to make sessions less content heavy and enable facilitators to attend to the core intervention principles (e.g. creating a positive atmosphere, encouraging sharing of experiences and highlighting similarities), as well as flexibility with delivery, to ensure intervention function is achieved	Changes to manual content including: Removing educational content about food groups from sessions (e.g. sections on Meat, Dairy, Fruit and Vegetables and the EatWell Plate) and moving to the Handbook. All group‐based activities and Behaviour Change Techniques were retained within sessions. More time dedicated to group activities (e.g. group pulse) that are designed to foster a sense of shared social identity and sharing of experiences
**Facilitator's confidence in delivering flexibly** (facilitator interviews, video/audio data and fidelity checklists)	To cut the amount of content needing to be delivered To aid flexibility of delivery and encourage delivery according to the group's needs. To aid facilitators' confidence in delivering flexibly	A priority key was introduced to indicate important activities, which are highly related to function (e.g. group pulse), as well as activities which can be tailored to the group's needs/knowledge
**Delivery according to PROGROUP principles** (audio and video data)	For use within intervention training, on how to action the core principles of the intervention	Development of a framework for delivery according to PROGROUP principles, specifying examples of good practice in facilitator delivery, from video and audio data. (see Supporting Information S9)
**Lack of patient engagement and connection within and outside of sessions** (patient interviews, end of intervention feedback, end of session feedback)	To foster a sense of ownership and leadership amongst group members	Assigning ‘champions’ to various activities, such as setting up/leading on a social media group, documenting the group's change and finding out about physical activity opportunities

## DISCUSSION

The function of the PROGROUP intervention was to build a sense of shared social identity to promote patient motivation and capability to engage in behaviour change, using core delivery principles, and fixed and flexible activities, designed to promote this function. Triangulation of quantitative and qualitative data from multiple sources demonstrated that for some groups, flexible delivery by facilitators of manual content according to the PROGROUP principles, led to achieving the function of the intervention. However, lack of flexibility in delivery was influenced by the number of activities facilitators had to cover each session, facilitator confidence in delivering groups and their rapport with their group. This was also reflected in patients' experiences of a sense of shared social identity, with some groups experiencing a greater sense of shared social identity than others, due to facilitator delivery and composition of the group. Assessing fidelity to delivery of content and core function of the PROGROUP intervention, through the synthesis of mixed‐methods data, provided key insights to refine and optimize the intervention.

Intervention fidelity assessment offers understanding of whether an intervention has been delivered as intended and is replicable (Toomey et al., 2020). However, previous research has emphasized the importance of fidelity to an intervention's core purpose or function (Esmail et al., 2020; Hill et al., 2020; Toomey et al., 2020). While for many behavioural interventions, delivery of content (fidelity to form) is sufficient to achieve the function of the intervention (Esmail et al., 2020); for group‐based interventions, such as PROGROUP, the interactions between group members and facilitators add variation and complexity to the intervention's causal pathway. For this reason, according to the Social Identity Model of Behaviour Change, the social identity‐based function (in order to support motivation and capacity to engage in behaviour change) of group interventions requires active management of group processes through flexible delivery of manual content in response to the needs of the group (Tarrant et al., 2020). Therefore, assessing the extent to which the current forms of the intervention (manual content and delivery principles) allow this flexibility and refining these accordingly was crucial in triggering the assumed causal pathways that lead to behaviour change. In this study, it was evident that different factors such as amount of content, facilitator confidence and rapport with the group determined how the forms were used to create a sense of shared social identity. This demonstrates the complex nature of group interventions, in which managing and responding to the needs of the group are essential to achieving desired outcomes. Through assessing delivery fidelity and whether the function of PROGROUP had been achieved, it was evident that some aspects of the delivery manual could be refined to guide facilitators, particularly those less confident in managing groups, towards a more flexible approach. Training, key in supporting facilitators, was also adapted, but this will be reported in detail elsewhere.

The patient perspective is key in assessing fidelity (Toomey et al., 2020) and enabling understanding of whether PROGROUP achieved its aim of developing a shared sense of social identity between group members. Corroborating Khan et al. (2020), these data highlighted that a sense of social identity varied across groups, due to the composition and attendance of the group, and was most evident in similar and well‐attending groups, when the facilitator provided a safe space to share experiences and similarities were emphasized. By contrast, groups with more diversity were more affected by low attendance, and as a result, delivery was not always aligned with the PROGROUP principles. This reiterates that while a sense of shared identity can occur naturally and is affected by composition of the group (e.g. age and personalities) and continuity of attendance, these factors are also heavily interlinked with active facilitation during delivery, to ensure function is achieved. This can include understanding the individuals within the group and planning management of similarities or diversity through the manual activities. Here, where similarities were emphasized, this contributed to an increased sense of group connection, with some evidence of this leading to behaviour change and shared social identity.

### Implications of the study

This study adds to the literature by demonstrating an approach to assessing fidelity within group‐based interventions. Through planned fidelity assessments and use of a variety of fidelity measures, it was possible to optimize the intervention, with careful consideration of how to optimize achieving the core function of the intervention. This approach therefore provides a blueprint for evaluations of any complex group intervention, regardless of populations, to understand fidelity and provide a method for optimizing components related to the group‐based function of the intervention. Additionally, findings related to facilitator delivery also had implications for re‐development of group‐based training for staff members, which could also prove useful for future facilitators of group‐based programmes (reported elsewhere).

### Strengths and limitations

The study has several notable strengths. Firstly, triangulation of mixed‐methods data was synthesized in a systematic way, incorporating a variety of sources, adding to the rigour of the process evaluation and optimization process. Secondly, use of a variety of fidelity measures enabled rigorous assessment of both delivery and receipt of the intervention to understand whether the causal pathways had been triggered and the function of the intervention had been achieved. This is important given such domains of fidelity have previously been poorly assessed and have previously only focussed on delivery (Toomey et al., 2020; Walton et al., 2017).

However, while rigorous methods were used, it is possible that these methods did not capture the experiences of participants who were less engaged in the group, which may also be reflected in and explain the poor response rates for the Group Processes Questionnaire. It was also not possible to elicit responses from those who had withdrawn from the study, whose experiences of the intervention may have been less positive than those who remained and possibly highlighted further areas to be optimized. The low response rates could also have been a result of patients being unsure of how to answer questions, although a member of the research team was available to complete questionnaires over the telephone if they wished. As changes were implemented as part of a feasibility trial, the impact of the changes made during optimization will be assessed during the next phase of the project. One important area for future focus will be on assessing the impact of some of the experiences here on the mechanisms and outcomes of the programme, for example, the influence of group stability, (i.e. the extent to which the group comprises the same people each week) on fidelity to function and thus group connection. This will be explored in detail in the main trial of the intervention.

## CONCLUSIONS

This study sought to optimize a complex group‐based intervention by bringing together data pertaining both to the intervention (facilitator) delivery and the experiences of those who received it (patients). The PROGROUP intervention aims to support behaviour change amongst people living with severe obesity through developing shared social identity leading to motivation and capability to engage in change. Because of this core function, it is essential that the ‘forms’ of the intervention (i.e. the manual content and delivery principles) enable and guide the facilitator to deliver the intervention flexibly in ways which meet the needs of the specific group. The multiple comprehensive fidelity assessments employed here enabled refinement of the intervention by prioritizing group‐based activities, group discussion and how a flexible approach could be achieved. This assessment also provides a blueprint for other group‐based interventions wishing to assess fidelity and ensure refinements made at a feasibility level optimize adherence to an intervention's core function. The optimized intervention is now being evaluated in a main trial.

## AUTHOR CONTRIBUTIONS

**Lily Hawkins:** Investigation; writing – original draft; writing – review and editing; formal analysis; resources. **Mark Tarrant:** Conceptualization; funding acquisition; writing – original draft; writing – review and editing; methodology; formal analysis; supervision. **Shokraneh Moghadam:** Investigation; writing – review and editing; resources; formal analysis. **Dawn Swancutt:** Funding acquisition; writing – review and editing; investigation. **Rod Sheaff:** Writing – review and editing; funding acquisition. **Laura Hollands:** Investigation; writing – review and editing. **Jonathan Pinkney:** Conceptualization; writing – review and editing; funding acquisition; supervision. **Jenny Lloyd:** Supervision; formal analysis; writing – review and editing; writing – original draft; methodology; funding acquisition.

## CONFLICT OF INTEREST STATEMENT

The authors declare that they have no conflict of interest.

## Supporting information


Data S1.

Data S2.

## Data Availability

Available upon request, following publication of the main trial findings.
