# Supplementary material for: Optimization of a group‐based intervention for people living with severe obesity (PROGROUP): Understanding fidelity to delivery and the patient experience
Source: Br J Health Psychol. 2026 Feb 2;31(1):e70051. doi: 10.1111/bjhp.70051 (PMC12862884; doi:10.1111/bjhp.70051)
Supplement: Supplementary file 2 — Data S2. [file BJHP-31-0-s001.docx]

**Supplementary material**

1. **The PROGROUP Principles**


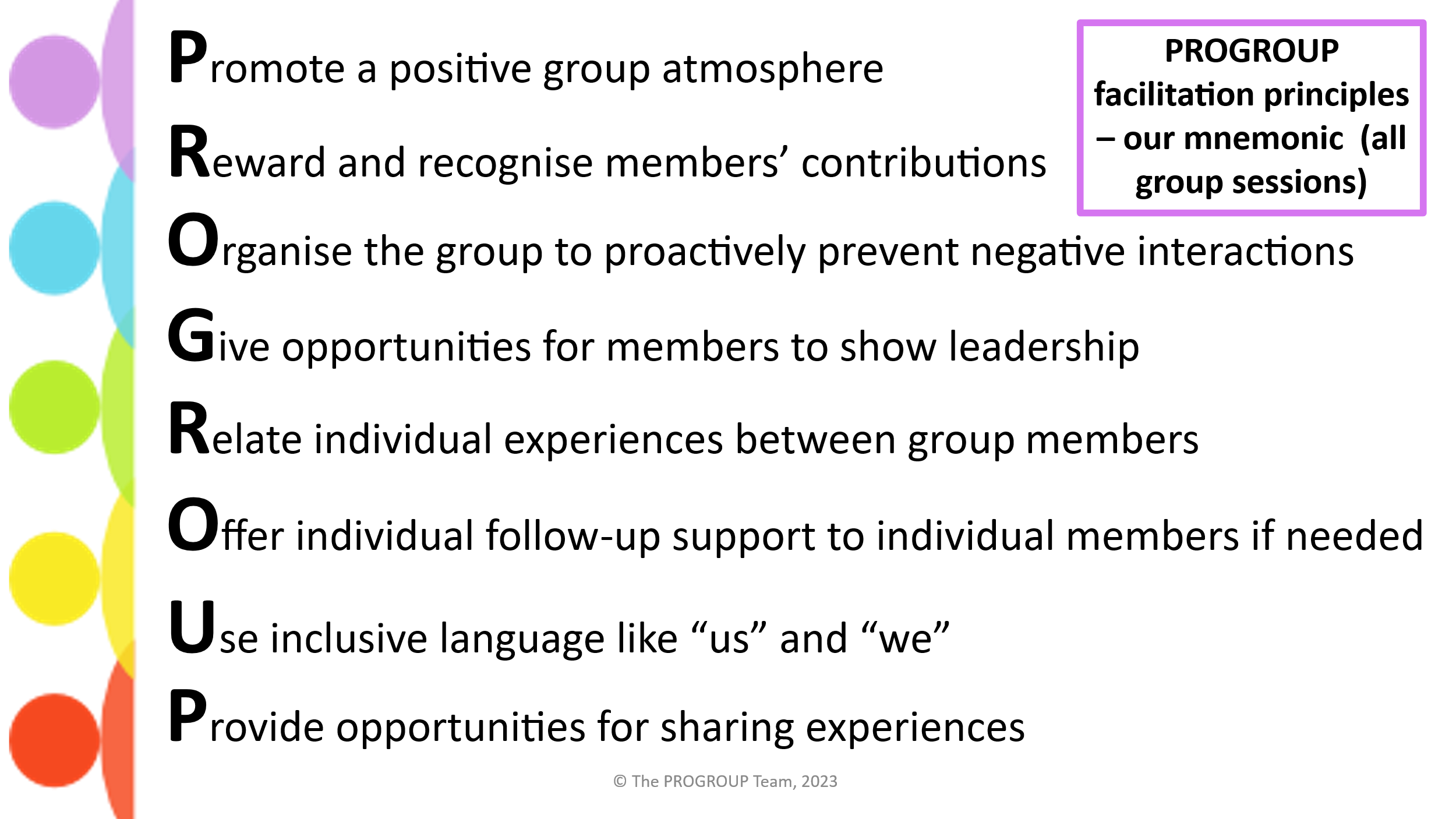


1. **Fidelity to manual content checklist (example)**

| *SITE ID:* | *FACILITATOR NAME:* | *DATE:* | | | *GROUP ID IF SITE RUNS MORE THAN 1 GROUP:* |
| --- | --- | --- | --- | --- | --- |
| **SESSION 9 – A HUNGRY BELLY HAS NO EARS** | | **PLEASE TICK** | | | **IF NOT DELIVERED OR PARTLY DELIVERED, PLEASE GIVE A BRIEF REASON FOR NOT DELIVERING** |
|  |  | ***FULLY DELIVERED*** | ***PARTLY DELIVERED*** | ***NOT DELIVERED*** |  |
| **START OF SESSIONS** | 1. **Recap from Session 8:**  Did you?   - Ask group about ‘lightbulb’ moment? |  |  |  |  |
| **KNOWLEDGE & ACTIVITIES** | 2. **Eating when not hungry**:  Did you?   - As a whole group, make a plan for one or two of the suggestions form the flipchart? - Allow group to choose which suggestion to focus on? |  |  |  |  |
|  | 3. **Session Break:**  Did you?   - Mix with members and facilitate conversation? |  |  |  |  |
|  | 4. **Eating Away from Home:**  Did you?   - Provide information on the topic of eating out and takeaways? - Ask group to discuss their experience via shout out? - Reassure group that making mistakes is ok but more healthy options are better? - Ask the group what their favourite takeaways/restaurants are? - In small groups, ask group to identify healthier options from their takeaway menus? - Share information on suggestions for common restaurant? - Share information on fake-aways? |  |  |  |  |
|  | 5. **Group Pulse**  Did you?   - Encourage group members to reflect on their progress – what is going well / not so well? |  |  |  |  |
| **END OF SESSION** | 6. **End of Session**  Did you?   - Outline homework tasks and ask group to shout-out take-home messages? |  |  |  |  |
| **GENERAL COMMENTS:** Please write any general comments you have about today’s session in the box below | | | | | |
|  | | | | | |

| **In today’s session, you may have carried out some specific actions/activities to ensure the group functioned well. Please indicate in the table below whether you carried out any of the following:** | | |
| --- | --- | --- |
|  | YES | NO |
| Actions/activities to manage negative group interactions, or manage dominant group members |  |  |
| Actions/activities for individual members to take on leadership roles within the group |  |  |
| Actions/activities to encourage the group members to support each other (e.g., following expression of a negative experience) |  |  |
| Actions/activities to encourage the group members to praise/celebrate/validate each other’s positive experiences |  |  |
| Please write any comments you have about the actions/activities listed above here: | | |

**Please indicate which picture best describes your perception of how well connected, in general, the attendees of today’s session appeared to be. For example, Picture 1 would suggest that there was very little connection between group members – that they did not come together as a group at all. Whereas Picture 3 would suggest that group members were very closely connected – they very much came together as a group.**

1. **Fidelity to PROGROUP Principles checklist (example)**

| SITE ID: **[Anon]** | SESSION NUMBER: **9 AUDIO** | DATE: | GROUP ID IF SITE RUNS MORE THAN 1 GROUP: |
| --- | --- | --- | --- |
| **FACILITATOR [Anon]** | | | |
| Please comment on the extent to which the facilitator carried out the following behaviours within the entirety of this session (i.e., from beginning to end): | | | |
| 1. **Promoted** a positive group atmosphere (e.g., welcomed participants, use of humour and friendly language, provided clarification) | | Welcomes, use of humour, very friendly, good energy at beginning to get back into it. Feels like more of a conversation. Regularly talks things through and clarifies things with Ps.  Positivity of group noted by participant. | |
| 2. **Rewarded** and recognised members’ contributions (e.g., congratulated individuals and/or group on achievements, praised individuals and/or group contributions) | | Aspects right, needs to do this more explicitly. Rewards with constructive feedback but could work more on explicit praise e.g. ‘Well done on..’ or ‘that’s great….’  Occasionally praised individuals and group on their shared changes/contributions and highlighted how it was a change (constructive feedback). | |
| 3. **Organised** the group to proactively prevent negative interactions (e.g., identified and agreed to group rules, negotiated and managed group roles and expectations) | | Could stepped in more to manage dominant members  Overall there weren’t any negative interactions  Re-emphasised group rules for Whatsapp group | |
| 4**. Gave** opportunities for members to show leadership (e.g., encouraged group members to take lead in group discussions/tasks) | | Some voluntarily spoke and took leadership but could invite others to speak during activities – e.g. does anyone have anything to add? Or could’ve linked back to previous sessions, or asked them to break into groups to discuss ideas  [Facilitator] asked one participant to ensure everyone is added to Whatsapp group - leadership | |
| 5. **Related** individual experiences between group members (e.g., emphasised similarities between group members, encouraged group problem solving) | | Could’ve done a lot more of this – some missed opportunities to draw on similarities and compare experiences between members/individuals e.g. when going through eating away from home exercise. Often heard a lot about individual’s experiences but these experiences weren’t related back to other Ps.  But related similarities from 1:1s to all participants (that many were anxious in the beginning) and demonstrated how Ps have changed | |
| 6. **Used** inclusive language like “us” and “we” | | Feel of inclusivity comes across strongly and no major issues with this. Perhaps could’ve been more inclusive when acknowledging group’s contributions and kept extending this out to other Ps (e.g. buffet section) or ask for similar experiences | |
| 7. **Provided** opportunities for sharing experiences (e.g., invited group members to share experiences, asked for group and/or individual feedback) | | Lots of discussion between group members and regularly invited members to share experiences for each exercise.  But could’ve provided more gaps/paused more to see if quieter members would contribute, or prompted less dominant members to discuss  Harder when there’s a dominant member  Could’ve drawn on small groups more and individual feedback | |
| **Additional Notes (e.g., contextual detail)** | | | |
| - **Overall great but still needs work to reinforce PROGROUP principles** - Discussed Wegovy for a long time. Could’ve been shut down quicker and related back to why learning behavioural strategies is also important (role of PROGROUP) - Did focus on some individuals a bit too much, didn’t always relate back - Group pulse – they seem to be a well formed group and have good cohesion. [Facilitator] offered own ideas (almost as though part of the group). | | | |

**4) Interview topic guides**

**a) Facilitator interview topic guide**

**PROGROUP Feasibility RCT Facilitator Interview Topic Guide (intervention only)**

Introductions and confirmation of consent process and voluntary nature of the research.

1. **Background**

*Aims: - To get facilitator talking and to provide some context for subsequent questions/responses.*

Could you tell me a little about your history of involvement in WM programmes

Probe on facilitation experience, role within the service.

1. **Experiences of PROGROUP training**

*Aims: To understand how acceptable the training programme is for facilitators - their engagement with the content and their confidence to deliver PROGROUP.*

Could you tell me how you found the PROGROUP training?

Probe on how they felt before, during and after the training and their experience of the content/activities/delivery.

​​     ​

1. **Experiences of delivering PROGROUP**

*Aims: To explore aspects of delivery – what worked, what didn’t work so well, delivery challenges, how challenges were overcome etc.*

Could you tell me how delivery of PROGROUP went for you?

Probe whether experiences changed over time (beginning, middle and end), what worked, what was challenging, how they overcame these, management of group processes.

Could you tell me if there any staffing issues in relation to delivering PROGROUP?

Probe whether they also delivered usual care, whether the commitment to delivering PROGROUP took them away from other duties, and, if so, the impact of this.

Could you tell me how you think the participants responded to PROGROUP?

Probe levels of engagement, attrition

1. **Acceptability of Trial Processes**

*Aims: To understand facilitator experience of being involved in the trial.*

Could you tell me how you felt about being involved in the trial?

Probe the quality of interactions with colleagues/researchers, fears/concerns/issues and whether they were sufficiently allayed.

How did you feel about completing checklists on the content you delivered each session?

Probe whether they actually did this, when they completed the forms and how much of a burden these were.

How did you feel about having your session audio/video recorded?

Probe whether this impacted the delivery of the session in any way.

**5. Contamination**

*Aims: To ascertain if facilitators who also deliver usual care import aspects of PROGROUP*

Did you use aspects of PROGROUP when you delivered usual care?  If so, what were they?

Probe which aspects and why they chose these.

1. **Suggestions for improving PROGROUP and the wider service**

*Aim: To support the optimisation of PROGROUP and the Tier 3 WM service*

What would you change about PROGROUP to maximise the support for change that patients receive?

Probe why these refinements would help

What would you change about training to help you deliver PROGROUP?

Probe why these refinements would help

Have you any comments/thoughts about how the wider service could be improved?

Would you like to share anything else about your experiences?

**b) Patient interview topic guide**

**PROGROUP Feasibility RCT Patient Interview Topic Guide (intervention only)**

Introductions and confirmation of consent process and voluntary nature of the research.

1. **Background**

*Aims: - To get patient talking and to provide some context for subsequent questions/responses.*

Could you tell me what brought you to this programme?

Probe on other services attended, adherence/attrition, external factors influencing weight management, maintenance of behaviours related to weight, social support, social participation, experience of stigma.

1. **Experiences of PROGROUP**

*Aims: To understand how acceptable the programme is for patients - their engagement with activities, facilitator and peer to peer support, opportunities, motivation, and capability to make and sustain changes.*

Could you start by telling me about your first group session and your first 1-1 session.  Shall we start with the 1-1 session?

Probe on how they felt before, during and after the session and their experience of the content/activities/facilitation.

Probe accessibility (e.g. getting there/parking and of the materials)

Probe how ‘invited’ they felt to attend (feeling safe, valued etc)

Ok let’s move on to the following sessions (I can remind you of these if that helps). How did these go for you?

Probe on:

Opportunities given to bond with group members, reflect on and discuss issues/barriers to weight management, problem solve, set group goals, to provide peer support.

Receipt of support from peers

How connected they felt to the group

Use of handouts/information/homework

Motivation and capability to make changes (personal and group goals) – link to facilitation and programme content

 ​     ​

1. **Behaviour Change**

*Aims: To explore the behavioural impact of PROGROUP and any barriers to making changes*

Could you tell me a little about any changes you have made because of the programme?

Probe physical activity, eating behaviours, family changes, social participation (i.e. doing community things)

Probe when in the programme they made these changes

Probe if any of these changes were part of a group goal, and, if so, did this make it easier/harder to achieve.

Could you tell me about any barriers you might have experienced in making changes?

Probe what helped to overcome/mitigate these barriers

Probe what strengthened these barriers

1. **Acceptability of Trial Processes**

*Aims: To understand patient experience of recruitment, randomisation and completion of measures.*

Could you tell me about your experience of being recruited into the study?

Probe the quality of interactions with staff/researchers, fears/concerns/issues and whether they were sufficiently allayed.

How did you feel about being assigned to PROGROUP rather than usual care?

Probe whether this affected their motivation during the programme

What did you feel about the study questionnaires you were asked to complete?

Probe information on each type of measure (participant characteristics, outcome, process and resource use) and how much of a burden these were.

**5.     Contamination**

*Aims: To ascertain if patients discussed* PROGROUP and/or shared PROGROUP resources with non-intervention participants)

Did you discuss PROGROUP/share resources with other patients outside of your group?

Probe which patients and what they discussed/shared and on how many occasions.

1. **Suggestions for improving PROGROUP and the wider service**

*Aim: To support the optimisation of PROGROUP and the Tier 3 WM service*

If you would change anything about PROGROUP to give you a better experience, what would that be?

Probe why these refinements would help.

What changes (if any) would you help you make lifestyle changes that you could continue after the programme.

Probe why these refinements would help.

Have you any comments about the service more generally?

**5) End of session/intervention questionnaires**

**a) Patient end of session feedback form (example)**

**PROGROUP SESSION FEEDBACK FORM FOR PARTICIPANTS**

| **Session 2** | **Group** | **Site 1** |
| --- | --- | --- |

**We would like to know what you think about this session and would be grateful for your feedback. The feedback is anonymous, and it will help us refine and improve the programme in future.**

1. **Overall, how satisfied are you with the session? (please circle one)**

| **Not at all satisfied** |  |  |  | **Very satisfied** |
| --- | --- | --- | --- | --- |
| **1** | **2** | **3** | **4** | **5** |

1. **How much do you agree with the following statement?**

**Today, the group members felt tense and anxious (please circle one)**

| **Not at all** |  |  |  |  |  | **Extremely** |
| --- | --- | --- | --- | --- | --- | --- |
| **1** | **2** | **3** | **4** | **5** | **6** | **7** |

1. **To what extent have you understood how to perform any of the strategies introduced in the session today? (please circle one)**

| **Not understood** |  |  |  |  |  | **Mostly understood** |
| --- | --- | --- | --- | --- | --- | --- |
| **1** | **2** | **3** | **4** | **5** | **6** | **7** |

1. **To what extent have you put into practice any of the strategies given in the sessions? (please circle one)**

| **Not at all** |  |  |  |  |  | **A lot** |
| --- | --- | --- | --- | --- | --- | --- |
| **1** | **2** | **3** | **4** | **5** | **6** | **7** |

1. **Which picture best describes how well connected, in general, you felt to the rest of the group today.**

**For example, the first picture would suggest that you felt very little connection to the rest of the group, whereas the final picture would suggest that you felt very connected to the group.**

**Please tick the box on the right-hand side of the picture that best describes how you felt.**

**
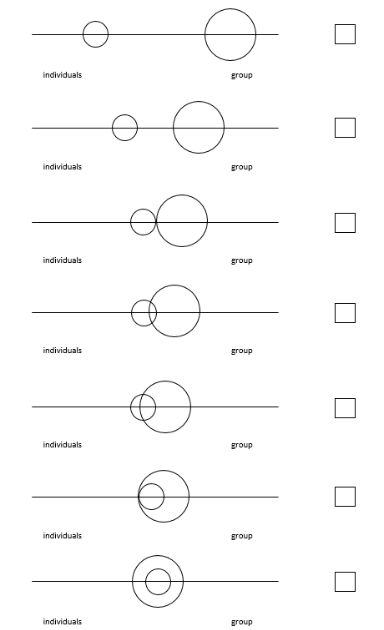
**

**b) End of intervention feedback**

**Introduction**

Thank you for taking part in the PROGROUP study. We would like you to complete the following questions about your experiences of the weight management service and the research study. People have said that it usually takes about 5-10 minutes to complete these questions. Please answer each question carefully. Your answers are very important to the study.

1. **How did you access the programme? (please circle one)**

- Face to face
- Online
- A mix of both face to face and online

1. **Did you withdraw from the weight management programme? (i.e. stop attending scheduled sessions or clinical appointments)**

Yes/No

If yes, when in your programme did you withdraw?

Month / Year ____

What was the reason for this?

1. **Did you miss any scheduled programme sessions (e.g. group meetings, clinical appointment etc?**

Yes/No

If yes, How many?

What was the reason for this?

1. **Overall, how satisfied are you with the weight management service? (please circle one)**

Not at all satisfied 1 2 3 4 5 Very satisfied

1. **Would you recommend the service to other patients?**

Not at all 1 2 3 4 5 Very much

1. **We would like to know if you have had contact with members of the weight management programme outside scheduled appointments/sessions?** Please tick all options.

|  | **Often** | **Sometimes** | **Once or twice** | **Never** |
| --- | --- | --- | --- | --- |
| **Written message (e.g. text, email, WhatsApp, Facebook)** |  |  |  |  |
| **Via a distanced conversation (e.g. phone, Zoom)** |  |  |  |  |
| **Meeting in person** |  |  |  |  |

1. **Please tell us what lifestyle changes you have made as a result of taking part in the weight management programme. Lifestyle changes can include anything you have changed and are continuing that helps you to manage your weight e.g. connecting more with your friends/eating at the table with the family/eating a healthy breakfast/walking in the park/monitoring your exercise etc.**

Please list the changes that have been **particularly helpful for you personally** in managing your weight

| Lifestyle change |
| --- |
|  |
|  |
|  |
|  |
|  |
|  |
|  |

1. **To what extent do you consider the following to be important to you personally? Circle the importance of each.**

1. Getting enough sleep

Not at all important 1 2 3 4 Very important

1. Being physically active

Not at all important 1 2 3 4 Very important

1. Eating fruit and vegetables

Not at all important 1 2 3 4 Very important

1. **To what extent do you agree with the following statement:**

**I feel anxious about my weight because of what people might think of me**

Strongly disagree 1 2 3 4 5 6 7 Strongly Agree

1. **FOR PROGROUP ARM PARTICIPANTS ONLY:**

**Did you discuss PROGROUP and/or share resources with other patients in the weight management service?**

Yes/No

If yes, were these patients in your PROGROUP group?

Yes/No

If no, what did you discuss/share?

**6) Group Processes Questionnaire**

**PROGROUP Questionnaire Pack: Participant experiences of the PROGROUP group programme**

**Introduction**

Thank you for taking part in the PROGROUP study. We would like you to complete the following questionnaire pack. There are four sets of questions complete.

People have said that it usually takes about 5-10 minutes to complete these questions. Some questions might seem obvious or repetitive but please answer each one carefully. Your answers are very important to the study.

 For each question, please circle the number on the scale which best represents how you feel

 The questions in this pack are about your experiences to date of the PROGROUP group programme provided by your specialist weight management service. By ‘group programme’ we mean the scheduled group sessions that you attend with other patients registered with the service.

**1. These questions are about your membership of the PROGROUP group**

**1a) I identify with members of the group**

| **Strongly disagree** |  |  |  |  |  | **Strongly agree** |
| --- | --- | --- | --- | --- | --- | --- |
| **1** | **2** | **3** | **4** | **5** | **6** | **7** |

**1b) I see myself as a member of the group**

| **Strongly disagree** |  |  |  |  |  | **Strongly agree** |
| --- | --- | --- | --- | --- | --- | --- |
| **1** | **2** | **3** | **4** | **5** | **6** | **7** |

**1c) I am pleased to be a member of the group**

| **Strongly disagree** |  |  |  |  |  | **Strongly agree** |
| --- | --- | --- | --- | --- | --- | --- |
| **1** | **2** | **3** | **4** | **5** | **6** | **7** |

**1d) I feel strong ties with members of the group**

| **Strongly disagree** |  |  |  |  |  | **Strongly agree** |
| --- | --- | --- | --- | --- | --- | --- |
| **1** | **2** | **3** | **4** | **5** | **6** | **7** |

**1e) Do you get the emotional support you need from other members of the group?**

| **Not at all** |  |  |  |  |  | **Definitely** |
| --- | --- | --- | --- | --- | --- | --- |
| **1** | **2** | **3** | **4** | **5** | **6** | **7** |

**1f) Do you get the help you need from other members of the group?**

| **Not at all** |  |  |  |  |  | **Definitely** |
| --- | --- | --- | --- | --- | --- | --- |
| **1** | **2** | **3** | **4** | **5** | **6** | **7** |

**1g) Do you get the advice you need from other members of the group?**

| **Not at all** |  |  |  |  |  | **Definitely** |
| --- | --- | --- | --- | --- | --- | --- |
| **1** | **2** | **3** | **4** | **5** | **6** | **7** |

**1h) To what extent do you think that being a member of the group helps you feel confident in your ability to manage your weight?**

| **Not at all** |  |  |  |  |  | **Very much** |
| --- | --- | --- | --- | --- | --- | --- |
| **1** | **2** | **3** | **4** | **5** | **6** | **7** |

**2. These questions are about your PROGROUP group facilitator(s) (for example, the dietician or other health care professional who runs your PROGROUP group)**

**2a) The facilitator(s) promotes the interests of members of the group**

| **Not at all** |  |  |  |  |  | **Completely** |
| --- | --- | --- | --- | --- | --- | --- |
| **1** | **2** | **3** | **4** | **5** | **6** | **7** |

**2b) The facilitator(s) acts as a champion for the group**

| **Not at all** |  |  |  |  |  | **Completely** |
| --- | --- | --- | --- | --- | --- | --- |
| **1** | **2** | **3** | **4** | **5** | **6** | **7** |

**2c) The facilitator(s) makes people feel as if they are part of the same group**

| **Not at all** |  |  |  |  |  | **Completely** |
| --- | --- | --- | --- | --- | --- | --- |
| **1** | **2** | **3** | **4** | **5** | **6** | **7** |

**2d) The facilitator(s) creates a sense of cohesion, or togetherness, within the group**

| **Not at all** |  |  |  |  |  | **Completely** |
| --- | --- | --- | --- | --- | --- | --- |
| **1** | **2** | **3** | **4** | **5** | **6** | **7** |

**2e) The facilitator(s) encourages interaction between the group members**

| **Not at all** |  |  |  |  |  | **Completely** |
| --- | --- | --- | --- | --- | --- | --- |
| **1** | **2** | **3** | **4** | **5** | **6** | **7** |

**2f) The facilitator(s) encourages cooperation between group members**

| **Not at all** |  |  |  |  |  | **Completely** |
| --- | --- | --- | --- | --- | --- | --- |
| **1** | **2** | **3** | **4** | **5** | **6** | **7** |

**2g) The facilitator(s) promotes a sense of trust between the group members**

| **Not at all** |  |  |  |  |  | **Completely** |
| --- | --- | --- | --- | --- | --- | --- |
| **1** | **2** | **3** | **4** | **5** | **6** | **7** |

**2h) The facilitator(s) helps the group members achieve their individual change goals**

| **Not at all** |  |  |  |  |  | **Completely** |
| --- | --- | --- | --- | --- | --- | --- |
| **1** | **2** | **3** | **4** | **5** | **6** | **7** |

**3. These questions are about the overall group atmosphere within your PROGROUP group**

**3a) The members liked and cared about each other**

| **Not at all** | **A little bit** | **Somewhat** | **Moderately** | **Quite a bit** | **A great deal** | **Extremely** |
| --- | --- | --- | --- | --- | --- | --- |
| **0** | **1** | **2** | **3** | **4** | **5** | **6** |

**3b) The members tried to understand why they do the things they do, tried to reason it out**

| **Not at all** | **A little bit** | **Somewhat** | **Moderately** | **Quite a bit** | **A great deal** | **Extremely** |
| --- | --- | --- | --- | --- | --- | --- |
| **0** | **1** | **2** | **3** | **4** | **5** | **6** |

**3c) The members avoided looking at important issues going on between themselves**

| **Not at all** | **A little bit** | **Somewhat** | **Moderately** | **Quite a bit** | **A great deal** | **Extremely** |
| --- | --- | --- | --- | --- | --- | --- |
| **0** | **1** | **2** | **3** | **4** | **5** | **6** |

**3d) The members felt what was happening was important and there was a sense of participation**

| **Not at all** | **A little bit** | **Somewhat** | **Moderately** | **Quite a bit** | **A great deal** | **Extremely** |
| --- | --- | --- | --- | --- | --- | --- |
| **0** | **1** | **2** | **3** | **4** | **5** | **6** |

**3e) The members depended upon the group leaders for direction**

| **Not at all** | **A little bit** | **Somewhat** | **Moderately** | **Quite a bit** | **A great deal** | **Extremely** |
| --- | --- | --- | --- | --- | --- | --- |
| **0** | **1** | **2** | **3** | **4** | **5** | **6** |

**3f) There was friction and anger between the members**

| **Not at all** | **A little bit** | **Somewhat** | **Moderately** | **Quite a bit** | **A great deal** | **Extremely** |
| --- | --- | --- | --- | --- | --- | --- |
| **0** | **1** | **2** | **3** | **4** | **5** | **6** |

**3g) The members were distant and withdrawn from each other**

| **Not at all** | **A little bit** | **Somewhat** | **Moderately** | **Quite a bit** | **A great deal** | **Extremely** |
| --- | --- | --- | --- | --- | --- | --- |
| **0** | **1** | **2** | **3** | **4** | **5** | **6** |

**3h) The members challenged and confronted each other in their efforts to sort things out**

| **Not at all** | **A little bit** | **Somewhat** | **Moderately** | **Quite a bit** | **A great deal** | **Extremely** |
| --- | --- | --- | --- | --- | --- | --- |
| **0** | **1** | **2** | **3** | **4** | **5** | **6** |

**3i) The members appeared to do things the way they thought would be acceptable to the group**

| **Not at all** | **A little bit** | **Somewhat** | **Moderately** | **Quite a bit** | **A great deal** | **Extremely** |
| --- | --- | --- | --- | --- | --- | --- |
| **0** | **1** | **2** | **3** | **4** | **5** | **6** |

**3j) The members rejected and distrusted each other**

| **Not at all** | **A little bit** | **Somewhat** | **Moderately** | **Quite a bit** | **A great deal** | **Extremely** |
| --- | --- | --- | --- | --- | --- | --- |
| **0** | **1** | **2** | **3** | **4** | **5** | **6** |

**3k) The members revealed sensitive personal information or feelings**

| **Not at all** | **A little bit** | **Somewhat** | **Moderately** | **Quite a bit** | **A great deal** | **Extremely** |
| --- | --- | --- | --- | --- | --- | --- |
| **0** | **1** | **2** | **3** | **4** | **5** | **6** |

**3l) The members appeared tense and anxious**

| **Not at all** | **A little bit** | **Somewhat** | **Moderately** | **Quite a bit** | **A great deal** | **Extremely** |
| --- | --- | --- | --- | --- | --- | --- |
| **0** | **1** | **2** | **3** | **4** | **5** | **6** |

**4. These questions are about the different social groups that you may belong to outside of PROGROUP. These could be leisure groups (e.g., book club, gardening club), community groups (e.g., church group), activity groups (e.g., walking group), work groups (e.g., sales team), or any other group you can think of**

**4a) I belong to lots of different groups**

| **Do not agree at all** |  |  |  |  |  |  |  | **Agree completely** |
| --- | --- | --- | --- | --- | --- | --- | --- | --- |
| **1** | **2** | **3** | **4** | **5** | **6** | **7** | **8** | **9** |

**4b) I join in the activities of lots of different groups**

| **Do not agree at all** |  |  |  |  |  |  |  | **Agree completely** |
| --- | --- | --- | --- | --- | --- | --- | --- | --- |
| **1** | **2** | **3** | **4** | **5** | **6** | **7** | **8** | **9** |

**4c) I am friendly with people in lots of different groups**

| **Do not agree at all** |  |  |  |  |  |  |  | **Agree completely** |
| --- | --- | --- | --- | --- | --- | --- | --- | --- |
| **1** | **2** | **3** | **4** | **5** | **6** | **7** | **8** | **9** |

**4d) I have strong ties with lots of different groups**

| **Do not agree at all** |  |  |  |  |  |  |  | **Agree completely** |
| --- | --- | --- | --- | --- | --- | --- | --- | --- |
| **1** | **2** | **3** | **4** | **5** | **6** | **7** | **8** | **9** |

**7) Optimisation matrix (attached)**

**8) Results - Supporting data**

1. Delivery of intervention

*[Content and confidence]*

*1.1 “…in the first few weeks the sessions were overfilled so… can you cut some fat from PROGROUP? You probably can” F03*

*1.2 “they were quite big sessions and they were a bit, I kinda get what you were trying to do when you were like we’ll do a bit around healthy eating and a bit around physical activity… but it had an occasion to feel a bit bitty” F01*

*1.3 “I think it’s about knowing who you’ve got in your group, knowing what discussions have gone on in previous sessions, knowing where it’s worth revisiting something because it’s clearly important for a number of people versus knowing when ‘well for that subject I don’t think we can make much more progress on that’ and so let’s focus on something else instead” F04*

*[Rapport with the group]*

*1.4 Most of us felt less comfortable showing slides than talking, again because [of] just establishing that relationship. F04*

*1.5 “I’m all about the notion of really bringing the group together as one, kind of forming some sort of a bond, being very kind of on that front so… I would have messaged them all the week, or the week before [the session]” F03*

*1.6 “…so we went through and agreed a name and that got everybody together having a bit of a laugh- having a visible relax in the group and then to set that WhatsApp group some people – some of the members expressed that they did want to do it - everyone got into it, happily joined the group, that was a big turning point” F02*

2. Patient experience

*[Delivery of programme by facilitator]*

*2.1 “They were just very open, and you felt comfortable, well I felt very comfortable with them both.” P2054*

*2..2 “No – I mean (staff names) were fab, I never felt judged or they weren’t condescending – they just got it and it felt like we’d known them years. They were really nice.” P2031*

*2.3 “Found the coordinator of the group easy to get on with and found her to be very understanding I think…the group I was in all agreed she made the journey fun but very worthwhile” Participant, End of Intervention Feedback*

*2.4 “…set up social media chat groups for fellow patients to join from the beginning of the course not during the middle after a 4-week gap. Didn't work.” Participant, End of Intervention Feedback*

*2.5 “We didn’t get a WhatsApp group going until quite later on in the sessions, and I really feel that having right at the beginning would have really helped… I really feel that in the future that’s something that should be done on the first if not second session.” P2054*

*2.6 “perhaps [if] something was put in place, if it was once or twice after that we met, even without XX, that [facilitator] facilitated - that this is where you’re meeting up, it might have gone on a bit longer” P3020*

*[Composition of the group]*

*2.7 “But once I got to know everyone, I felt welcome. It was a lovely bunch, we all got on really, really well and we were all so different.” P2015*

*2.8 “there was one gentleman for example, who's felt very conscious about being in a group like that and wanted to sit apart from the main group for the first session. But second session, he felt comfortable enough to join the group which was a great indication of how welcoming people were and not judgemental you know, and all of that sort of thing.” P3009*

*2.9 “You know, if there’s something bothering them or they haven't had a good week or whatever it is, then that that was the ideal platform for them to vent their problems. We were the right crowd to listen, because we understood.” P3009*

*[Attendance]*

*2.10 “I think it did, I think it affected us with regards to having that security to – the only consistent person was the facilitator and one or two people who came every single week. I couldn’t make it every week, there was some weeks I missed because of other things so when I’d come in, initially I’d just come in and listen and then get to talking whereas other people would just come in and sit and listen and were very reluctant to speak probably because we hadn’t set up that initial group we should have got done on day 1.” P2052*

*2.11 “Just that obviously instil in people that I think you've got to commit and at least try and be there, the majority of it.* *You know, you're taking away what other people could take from it, because they’re missing your input.” P2057*

**9) Delivery according to PROGROUP principles framework (example)**

| **EXAMPLES OF HOW FACILITATOR DEMONSTRATES PROGROUP PRINCIPLES:** | |
| --- | --- |
| 1. **Promoted** a positive group atmosphere (e.g., welcomed participants, use of humour and friendly language, provided clarification) | - Welcomes, goes through apologies from group members with the group attending - Use of humour, very friendly and provides clarification throughout session, recap for those who couldn’t attend - Non-verbal communication - lots of nodding and smiling when group members were talking. - Mindful of set up of room, be part of the group e.g. sit with them in a circle - Use of activities and humour to create energy – e.g. PA activity in Session 2 - Let’s group discuss between themselves for a while and steps in when going on a tangent - Uses group name |
| 2. **Rewarded** and recognised members’ contributions (e.g., congratulated individuals and/or group on achievements, praised individuals and/or group contributions) | - Acknowledges contributions regularly – links back to what’s been learnt or covered before in relation to what P is saying – e.g. “it goes to show it’s influencing your behaviour, you’re seeking out information to help make your mind up” in relation to P contributing and saying they’ve been checking labels - Occasionally praised individuals e.g. “You’ve really embraced tracking…” and group on their shared changes/contributions and highlighted how it was a change (constructive feedback) but also adds explicit praise e.g. “super stuff…” - Has mini conversations with each member who contributes, demonstrates recognition of what they are saying - Corrected negative language and acknowledges the attempt at changing - ‘Brilliant…well done’; Regularly acknowledges contributions positively - ‘Thanks for that’; ‘You tried it, that’s the important thing’… - Identifies and acknowledges all the positive steps taken by each member/the group’s efforts – ‘it’s great, you’re trying’ |
| 3. **Organised** the group to proactively prevent negative interactions (e.g., identified and agreed to group rules, negotiated and managed group roles and expectations) | - Identified group rules in early sessions and group suggestions/rules about Whatsapp group - Used group pulse as an opportunity to problem-solve and give group a name, and set up Whatsapp group - Acknowledges participant who vocalises a tangent topic, let’s them speak but quickly goes back to topic at hand. |
| 4**. Gave** opportunities for members to show leadership (e.g., encouraged group members to take lead in group discussions/tasks) | - Offer opportunities to lead on setting up Whatsapp group/group product (More about giving responsibility to the group/members of the group) - One member leading on Facebook group and putting key content from sessions on group |
| 5. **Related** individual experiences between group members (e.g., emphasised similarities between group members, encouraged group problem solving) | - Related similarities from 1:1s to all participants (that many were anxious in the beginning) and demonstrated how Ps have changed - Allows participants to talk to each other about similar experiences and advise each other (e.g. not eating breakfast/monitoring steps/losing weight – positives and negatives) and then adds knowledgeable information for group to consider - Recapped on ‘top topics’ for the group e.g. monitoring steps/fluid intake – highlights shared goals - Asked the group to help suggest ways of overcoming negative self-talk – generated discussion of how many of them feel the same – and then generated discussion on how to begin overcoming this - Advised to talk to the group for support with taking control of one aspect in your life |
| 6. **Used** inclusive language like “us” and “we” | - e.g. ‘We had a good discussion on…’, ‘That’s what’s ahead of us..’ |
| 7. **Provided** opportunities for sharing experiences (e.g., invited group members to share experiences, asked for group and/or individual feedback) | - Regularly signposts and questions to keep discussion going and invites other group members to add to conversation whilst guiding with useful information. - Ask participants directly and instigates discussion e.g. ‘how has X gone over the last week/what’s gone well?’, ‘What do we think about X?’, or ‘X has shared about their experience with X, what’s gone well for everyone else?’ - Directly asked quieter sub-groups and went round each member/subgroup - One facilitator – split into sub-groups for recap so each person gets a chance to contribute and feedback ‘highlights’. Invites contributions after small group discussions |
| **Additional Notes (e.g., contextual detail)** | |
| - Highlights key points and resources if runs out of time on certain topics and explains skill/BCT behind it and benefit of it | |
